# Compensatory biomechanics and spinal loading during dynamic maneuvers in patients with chronic low back pain

**DOI:** 10.1007/s00586-022-07253-4

**Published:** 2022-05-20

**Authors:** Priya Nyayapati, Jacqueline Booker, Peter I-Kung Wu, Alekos Theologis, Lucas Dziesinski, Conor O’Neill, Patricia Zheng, Jeffrey C. Lotz, Robert P. Matthew, Jeannie F. Bailey

**Affiliations:** 1 Department of Orthopaedic Surgery, University of California, 95 Kirkham St., San Francisco, CA 94122 USA; 2 Albany Medical College, Albany, NY USA; 3 School of Medicine, University of California, San Francisco, CA USA

**Keywords:** Low back pain, Biomechanics, Motion analysis

## Abstract

**Purpose:**

This study explores the biomechanics underlying the sit-to-stand (STS) functional maneuver in chronic LBP patients to understand how different spinal disorders and levels of pain severity relate to unique compensatory biomechanical behaviors. This work stands to further our understanding of the relationship between spinal loading and symptoms in LBP patients.

**Methods:**

We collected in-clinic motion data from 44 non-specific LBP (NS-LBP) and 42 spinal deformity LBP (SD-LBP) patients during routine clinical visits. An RGB-depth camera tracked 3D joint positions from the frontal view during unassisted, repeated STS maneuvers. Patient-reported outcomes (PROs) for back pain (VAS) and low back disability (ODI) were collected during the same clinical visit.

**Results:**

Between patient groups, SD-LBP patients had 14.3% greater dynamic sagittal vertical alignment (dSVA) and 10.1% greater peak spine torque compared to NS-LBP patients (*p* < 0.001). SD-LBP patients also had 11.8% greater hip torque (*p* < 0.001) and 86.7% greater knee torque (*p* = 0.04) compared to NS-LBP patients. There were no significant differences between patient groups in regard to anterior or vertical torso velocities, but anterior and vertical torso velocities correlated with both VAS (*r* = − 0.38, *p* < 0.001) and ODI (*r* = − 0.29, *p* = 0.01). PROs did not correlate with other variables.

**Conclusion:**

Patients with LBP differ in movement biomechanics during an STS transfer as severity of symptoms may relate to different compensatory strategies that affect spinal loading. Further research aims to establish relationships between movement and PROs and to inform targeted rehabilitation approaches.

## Introduction

Low back pain (LBP) is a major health problem in the U.S, present in 19.6% of adults between 20 and 59 years old [1]. LBP is difficult to diagnose due to its diverse and multifaceted etiology [2]. Patient-reported outcomes (PROs) for pain, disability, and health-related quality of life reflect patient experience, yet are difficult to link to specific underlying pathology [3]. Rather than subjective PROs, objective measurements of biomechanical function may better clarify associations between functional disability and pathology. In particular, biomechanical compensation, which occurs when people with LBP alter their physical behavior, creates abnormal and ineffective movement patterns that may negatively impact spinal loading [4].

A common in-clinic functional test that can distinguish the effect of LBP on physical function is the sit-to-stand (STS) transfer [5]. Current STS protocols assess the duration for a patient to complete five repeated STS transfers. While many studies confirm that the effect of LBP significantly increases the STS time [6], only few explore the compensatory biomechanics adopted by LBP patients during the STS test. Prior research has shown that LBP patients had decreased velocity of the trunk and hip compared to controls in an STS test [7]. Patients with LBP also have decreased lumbar and hip mobility [8]. However, there has been no established consensus regarding motion of the lumbar spine, as there is disagreement between studies. It has been established that patients with LBP have changes in their kinematics compared to controls, but there is a lack of the literature addressing the specific kinematics in regard to an isolated STS movement, specifically regarding spinal loading and sagittal alignment.

In this study, we conducted non-invasive in-clinic motion assessments of chronic LBP patient groups and compared their compensatory biomechanical behaviors. Two distinct chronic LBP patient subgroups were included: non-specific low back pain (NS-LBP) patients and spinal deformity patients with LBP (SD-LBP). We assessed compensatory biomechanics using novel markerless depth mapping technology [10] and compared computed kinematics and kinetics of the trunk and lower extremities between groups and in relation to PROs. We hypothesized that compensatory biomechanical behaviors are adopted by both LBP patient groups, with more compromised kinematics and spinal loading occurring among patients with more severe symptoms. The purpose of this study is to investigate the biomechanics underlying the STS functional maneuver in chronic LBP patients in order to understand how different spinal disorders and levels of pain severity relate to unique compensatory biomechanical behaviors. This work stands to further our understanding of the relationship between spinal loading and symptoms in LBP patients and may inform targeted rehabilitation approaches for specific spinal disorders and patient populations.

## Methods

### Sample

With IRB approval, we collected in-clinic motion analysis and outcomes data from patients during routine clinical visits. This study includes NS-LBP patients and SD-LBP patients. NS-LBP patients had at least 6 months of LBP symptoms and no clear underlying condition responsible for pain symptoms. SD-LBP patients also had at least 6 months of LBP symptoms, but also had spinal deformity conditions including adult degenerative scoliosis and hyperkyphosis and presented sagittal imbalance of at least 40 mm on standing radiography. Subjects were excluded if they had pain or dysfunction in the cervical spine or thoracic spinal regions. Additionally, subjects were excluded if they had unrelated pain or dysfunction in the lower extremities. All of the subjects were able to walk independently and perform an unassisted STS maneuver.

### In-clinic motion analysis and biomechanical modeling

Patients were asked to complete a maximum of nine unassisted STS maneuvers (three separate trials of three continuous maneuvers each). An RGB-depth camera (Kinect 2, Microsoft, Inc.) was placed in the frontal view and tracked 3D joint positions. Joint location estimates were filtered using an unscented Kalman filter (UKF) and an allometrically scaled, patient-specific rigid body model. The computed kinematic, kinetic, and dynamic parameters were then used to estimate maximum torque at the L5-S1 using a sagittal plane model of intra-abdominal pressure and the spine extensors. The acquisition of STS kinematic, kinetic, and dynamic metrics from the depth camera system has been validated [9, 10].

### Outcomes

**Fig. 1:**
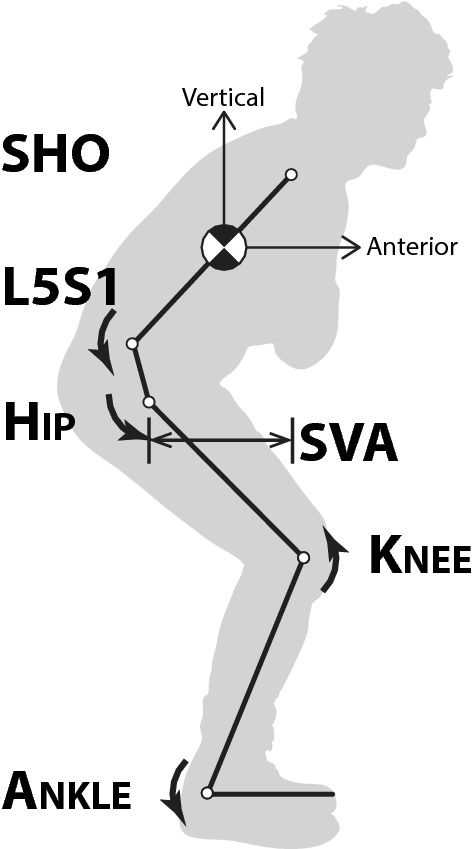
Joint centers sensed and processed to obtain kinematic, dynamic, and biomechanical metrics

The kinematic, kinetic, and dynamic metrics obtained from the STS were total time, peak excursion of normalized sagittal vertical alignment (dynamic SVA, dSVA; dimless), normalized peak anterior and vertical velocities for the torso (1/s), and normalized peak torques (dimless) at L5S1 and the hip and knee joints (Fig. 1). These variables are normalized by height measurements and are mostly dimensionless (dimless). dSVA is defined as the peak sagittal distance between the hip and shoulder centers and uses height as its scaling variable. The anterior and vertical torso velocities are the peak velocities of the torso in those directions. In addition, we collected patient-reported outcomes for back pain using the visual analog scale (VAS) (0–10) and for low back disability using the Oswestry Disability Index (ODI) (0–100) attained during the same clinical visit. For analysis, patients were grouped by low VAS (≤ 5.0) and high VAS (> 5.0) and by low ODI (≤ 50) and high ODI (> 50).

### Statistical analyses

STS data used for statistical analysis were averaged over multiple STS trials per subject. Between NS-LBP and SD-LBP patient groups, biomechanical variables and differences between patient groups in terms of VAS and ODI were compared using unpaired *t* tests. Significance was based on *p* < 0.05. All statistical analyses were done using R 1.1.463 (RStudio, Boston, MA, USA).

## Results

This study examines 86 subjects, including 44 NS-LBP patients (mean age: 54.1 ± 17.4) and 42 SD-LBP patients (mean age: 62.9 ± 11.9; Table 1). For the two patient groups pooled, mean VAS was 5.69 (± 2.84), and mean ODI was 50.0 (± 16.2). Mean VAS was significantly lower for the NS-LBP group (4.62 ± 2.36) compared to the SD-LBP group (6.91 ± 2.89; *p* < 0.001). Mean ODI was not significantly different between the NS-LBP (50.7 ± 16.5) and SD-LBP (49.1 ± 16.1) groups.

**Table 1:** Demographics, VAS, and ODI for each group

	Count	Age (years)	Sex	VAS	ODI
Total	86	58.39 ± 15.50	35 M, 51 F	5.69 ± 2.84	50.0 ± 16.2
NS-LBP	44	54.11 ± 17.35	22 M, 22 F	4.62 ± 2.36	50.7 ± 16.5
SD-LBP	42	62.88 ± 11.94	13 M, 29 F	6.91 ± 2.89	49.1 ± 16.1

### Peak torso velocity and dynamic sagittal balance

**Fig. 2:**
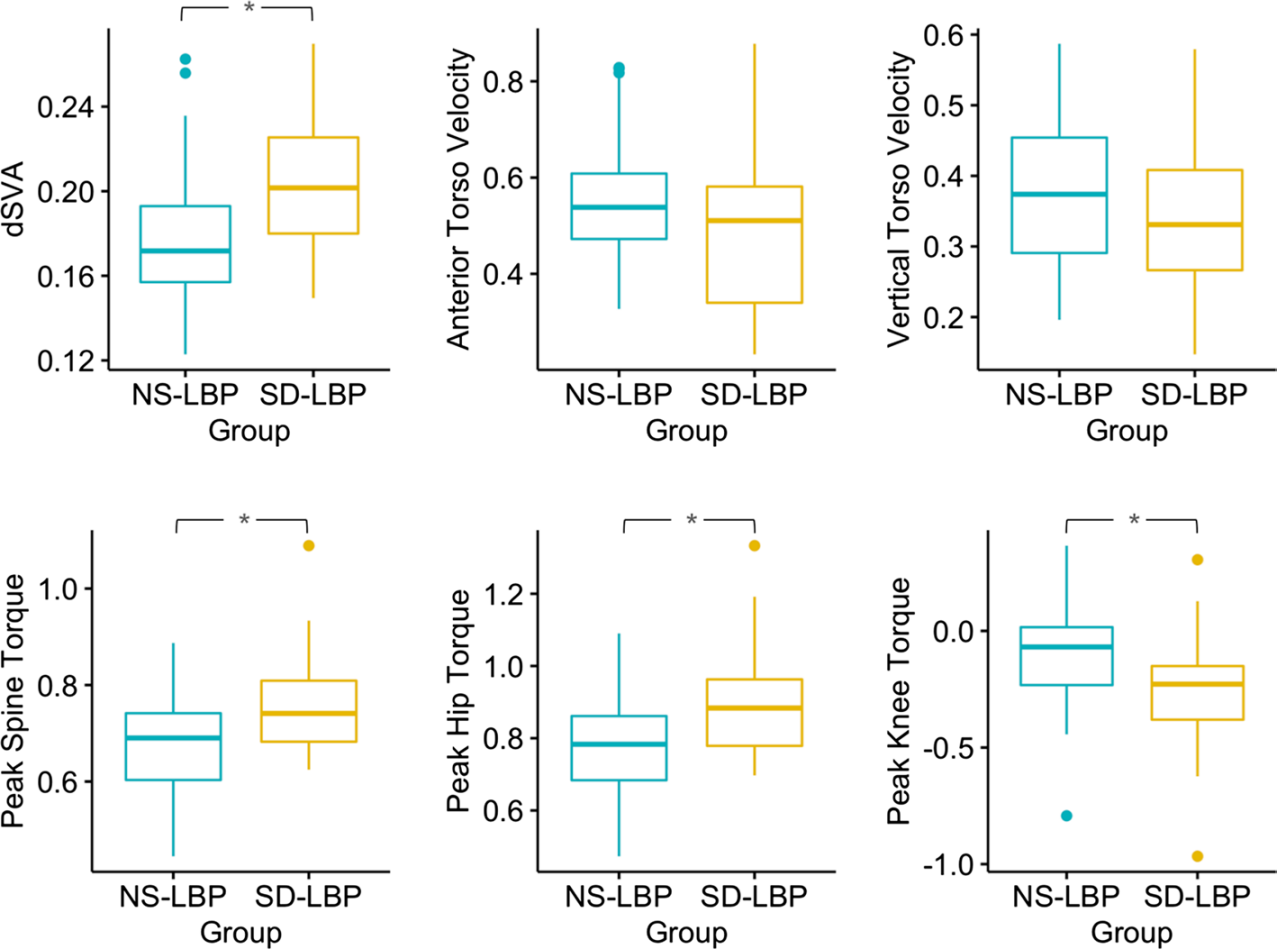
Box plots for between-group comparison in dSVA, torque, and torso velocities

There were differences between chronic LBP patient groups in regard to dynamic sagittal balance. dSVA for the SD-LBP patients was 14.3% greater compared to NS-LBP patients (*p* < 0.001, Table 2, Fig. 2). There were no differences in anterior or vertical torso velocity between patient groups.

**Table 2:** Normalized biomechanical metrics for controls, all LBP patients, NS-LBP patients, and SD-LBP patients and between-group comparisons

	All Patients	NS-LBP	SD-LBP	SD-LBP compared to NS-LBP
dSVA (dimless)	0.19 ± 0.03	0.18 ± 0.03	0.21 ± 0.03	14.3%, *p* < 0.001
Peak anterior torso velocity (1/s)	0.52 ± 0.15	0.6 ± 0.14	0.53 ± 0.16	n.s
Peak vertical torso velocity (1/s)	0.36 ± 0.11	0.4 ± 0.11	0.37 ± 0.12	n.s
Max L5S1 flexion torque(dimless)	0.71 ± 0.11	0.71 ± 0.11	0.79 ± 0.1	10.1%, *p* = 0.003
Max hip flexion torque (dimless)	0.83 ± 0.15	0.82 ± 0.15	0.93 ± 0.13	11.8%, *p* < 0.001
Max knee flexion torque (dimless)	− 0.17 ± 0.24	− 0.02 ± 0.25	− 0.15 ± 0.28	*p* = 0.04

**Fig. 3:**
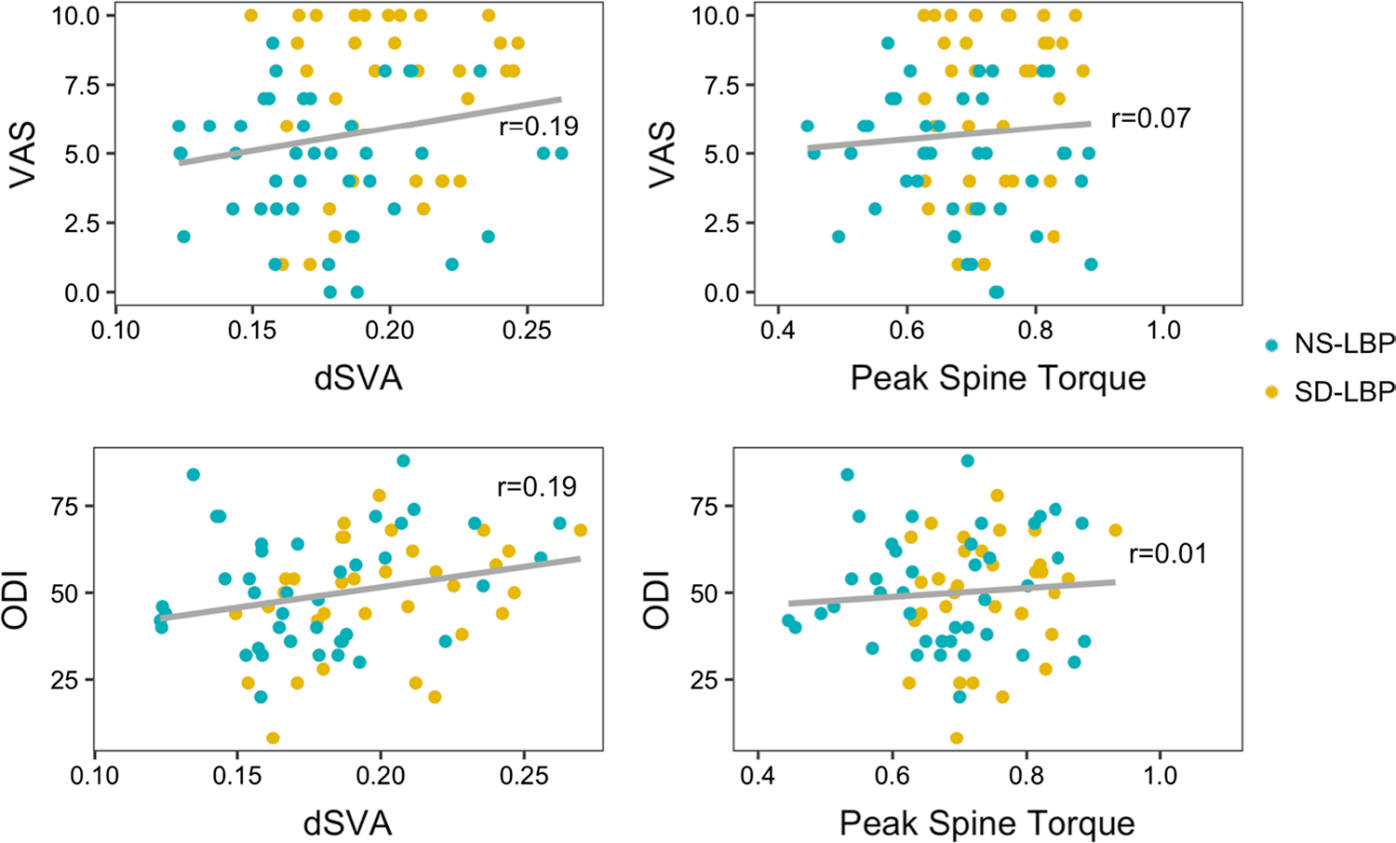
Correlations between dSVA and peak spine torque with VAS and ODI

**Fig. 4:**
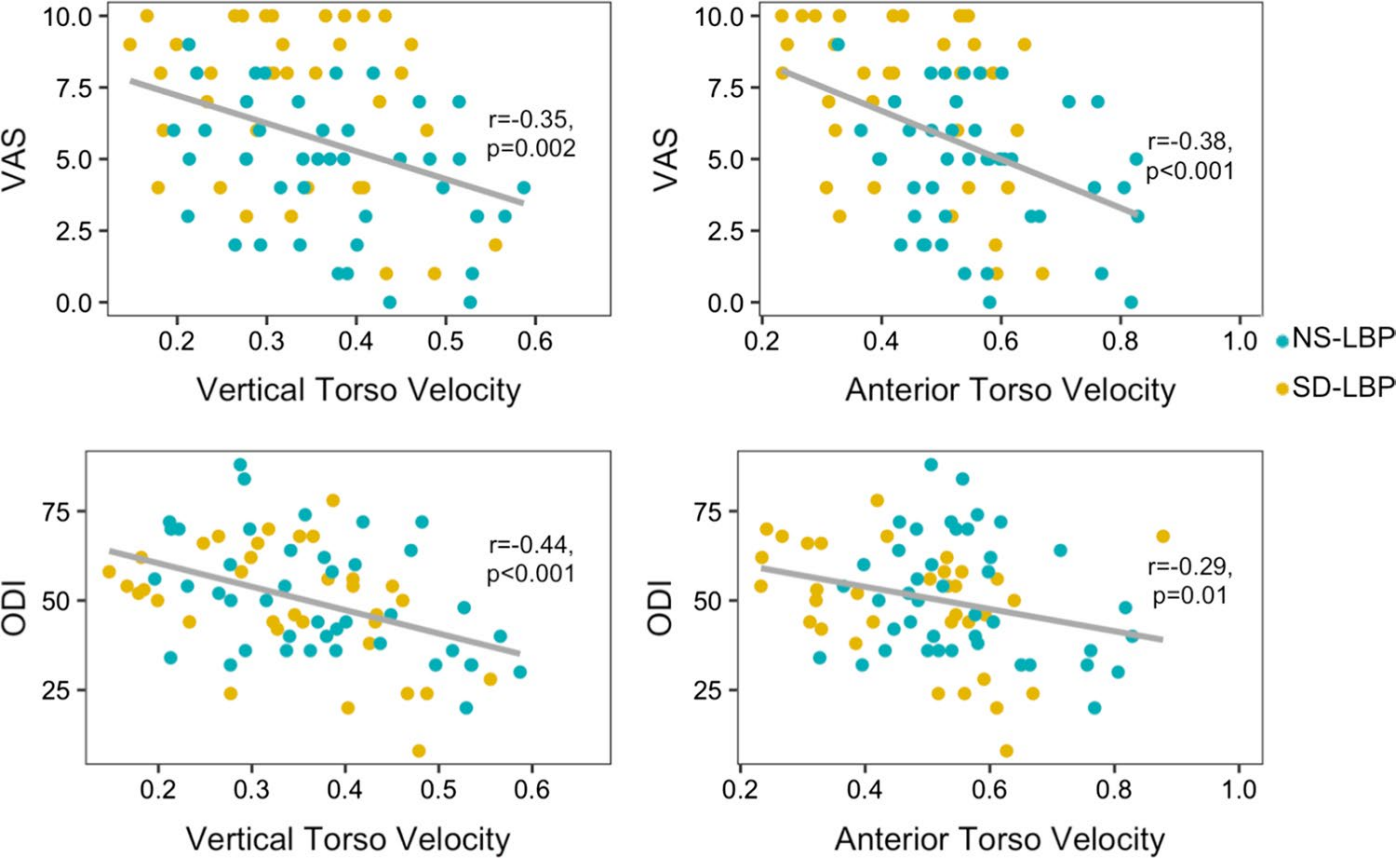
Correlations between torso velocity with VAS and ODI

There was no overall correlation between VAS and dSVA (Fig. 3; Table 3), but VAS negatively correlated with anterior torso velocity (*r* = − 0.38, *p* < 0.001, Fig. 4) and vertical torso velocity (*r* = − 0.35, *p* = 0.002; Fig. 4). Within the NS-LBP patient group, patients with high VAS had 20% lower vertical torso velocity compared to those with low VAS (*p* < 0.05, Table 3). Between patient groups, SD-LBP patients with low VAS had 16.7% lower anterior torso velocity compared to NS-LBP patients with low VAS (*p* = 0.04, Table 3).

**Table 3:** Normalized biomechanical data for NS-LBP and SD-LBP patients classified by low and high VAS

		NS-LBP	SD-LBP
	VAS	Mean ± SD	Mean ± SD
dSVA (dimless)	Low	0.19 ± 0.03	0.21 ± 0.02
	High	0.18 ± 0.03	0.21 ± 0.03
Peak anterior torso velocity (1/s)	Low	0.63 ± 0.15	0.57 ± 0.12
	High	0.56 ± 0.11	0.48 ± 0.15
Peak vertical torso velocity (1/s)	Low	0.42 ± 0.11	0.4 ± 0.15
	High	0.35 ± 0.1	0.34 ± 0.11
Max L5S1 flexion torque (dimless)	Low	0.73 ± 0.11	0.76 ± 0.09
	High	0.68 ± 0.1	0.78 ± 0.09
Max Hip flexion torque (dimless)	Low	0.84 ± 0.15	0.9 ± 0.11
	High	0.77 ± 0.14	0.92 ± 0.12
Max knee flexion torque (dimless)	Low	0.01 ± 0.24	0.001 ± 0.34
	High	− 0.07 ± 0.27	− 0.23 ± 0.24

There was no overall correlation between ODI and dSVA (*r* = 0.19, Fig. 3), but ODI negatively correlated with anterior torso velocity (*r* = − 0.29, *p* = 0.01, Fig. 4) and vertical torso velocity (*r* = − 0.44, *p* < 0.001; Fig. 4). For patients with low ODI, NS-LBP patients had a 23.5% lower dSVA compared to SD-LBP patients (*p* = 0.001). For NS-LBP patients, those with high ODI had 15% greater dSVA compared to those with low ODI (*p* = 0.02, Table 4). NS-LBP patients with high ODI also had 16.7% lower vertical torso velocity compared to NS-LBP patients with low ODI (*p* = 0.003, Table 4). For SD-LBP patients, those with high ODI had 31.3% lower vertical torso velocity compared to those with low ODI (*p* = 0.02, Table 4).

**Table 4:** Normalized biomechanical data for NS-LBP and SD-LBP patients classified by low and high ODI

		NS-LBP	SD-LBP
	ODI	Mean ± SD	Mean ± SD
dSVA (dimless)	Low	0.17 ± 0.03	0.21 ± 0.03
	High	0.2 ± 0.04	0.22 ± 0.02
Peak anterior torso velocity (1/s)	Low	0.63 ± 0.16	0.56 ± 0.15
	High	0.56 ± 0.09	0.49 ± 0.17
Peak vertical torso velocity (1/s)	Low	0.44 ± 0.11	0.42 ± 0.12
	High	0.34 ± 0.09	0.32 ± 0.11
Max L5S1 flexion torque (dimless)	Low	0.69 ± 0.12	0.78 ± 0.08
	High	0.73 ± 0.1	0.78 ± 0.09
Max hip flexion torque (dimless)	Low	0.78 ± 0.15	0.91 ± 0.11
	High	0.86 ± 0.14	0.93 ± 0.12
Max knee flexion torque (dimless)	Low	− 0.01 ± 0.21	− 0.05 ± 0.36
	High	− 0.06 ± 0.27	− 0.25 ± 0.21

### Peak load on the lower back

**Fig. 5:**
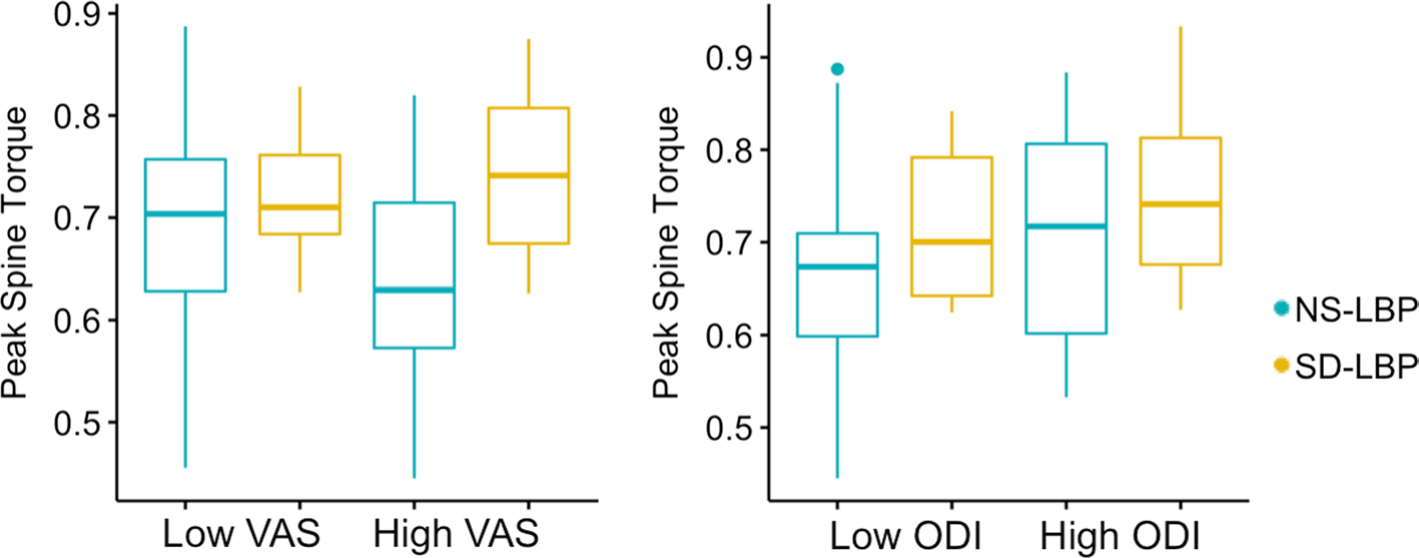
Box plots of between-group differences stratified by low and high VAS and ODI for peak spine torque

There were significant differences between patient groups for peak torque on the lower back. SD-LBP patients had 10.1% greater spine torque compared to NS-LBP patients (*p* = 0.003, Table 2, Fig. 2). There was no significant overall correlation between either VAS or ODI with spine torque (Fig. 3), but SD-LBP patients with high VAS had 12.8% greater spine torque compared to NS-LBP patients with high VAS (*p* = 0.002, Table 3, Fig. 5). Also, SD-LBP patients with low ODI had 11.5% greater spine torque than NS-LBP patients with low ODI (*p* = 0.01, Table 4, Fig. 5).

### Lower extremity

There were significant differences between patient groups in regard to peak hip and peak knee torque. SD-LBP patients had 11.8% greater peak hip torque compared to NS-LBP patients (*p* < 0.001). NS-LBP and SD-LBP patients had peak torque in the knee with flexion compared to extension. SD-LBP patients had 86.7% greater peak knee torque compared to NS-LBP patients (*p* = 0.04; Table 2, Fig. 2).

There was no overall correlation between VAS and hip torque, but in patients with high VAS, SD-LBP patients had 16.3% greater peak hip torque compared to NS-LBP patients (*p* = 0.002, Table 3). There was a significant correlation between VAS and knee torque (*r* = − 0.28, *p* = 0.01), but no differences within or between patient groups when stratifying by high/low VAS or ODI.

There was no significance in the overall correlation between ODI and hip torque (*r* = 0.09), but between patients with low ODI, SD-LBP patients had 14.3% greater peak hip torque compared to NS-LBP patients (*p* = 0.003, Table 4). There was a significant correlation between ODI and knee torque (*r* = − 0.23, *p* = 0.04). Between patients with high ODI, SD-LBP patients had greater knee extension torque compared to NS-LBP patients (*p* = 0.02; Table 4).

## Discussion

We observed that compensatory movements during STS differ between NS-LBP and SD-LBP patients. Dynamic SVA (dSVA) and spinal loading (peak spine torque) showed significant differences, as SD-LBP patients had 14.3% greater dSVA and 10.1% greater spine torque compared to NS-LBP patients, suggesting that spinal loading differs based on underlying disorder. Differences in hip and knee torque between patient groups also suggest different distributions of load on the lower extremities that may reflect overall compensatory strategies in response to different spinal conditions. Lastly, trunk kinematics and joint loading did not show a correlative relationship with patient-reported pain and disability across LBP patients, but we found that relationships between biomechanical metrics and PROs were specific to distinct LBP patient groups. Biomechanical compensation as it relates to pain and disability in LBP patients may account for underlying conditions and available diagnoses.

Associations between LBP and decreased trunk velocity and flexion have been well described in prior work [5, 11, 12]. The changes are thought to be adaptive movements to limit torso movement in order to avoid pain [13]. Performing dynamic movements using lower torso velocity seems to alleviate pain for LBP patients, but there has been no well-defined relationship established between torso velocity and pain scores or pathology. This suggests that torso velocity alone is not sufficient enough to differentiate spinal conditions or pain between patient groups and that there are other differences in the biomechanical movements between LBP patients, potentially reflected in spinal loading.

The SD-LBP and NS-LBP patient groups were distinguished by factors related to spinal loading, including increased dSVA and peak spine torque. Sagittal alignment is important to maintain balance without using an excess amount of energy, so an increase in SVA means increased work is required to maintain balance [14] and is associated with severity of LBP symptoms [15]. Realignment of SVA through thoracolumbar corrective fusion correlates with reduced disability [16], and dSVA improves with spinal realignment surgery in adult spinal deformity patients [9], suggesting that correction of SVA reduces loading on the lower lumbar spine. Placing increased torque on the lumbar spine repetitively may be associated with worse long-term outcomes [17], even if surgery is performed [18]. Implications of these findings raise concern that compensatory movements that increase torque on the spine may contribute to the progression of spinal deformity. Further exploration of the relationship between spinal load and dSVA and the potential structural consequences may provide more insight into predicting surgical candidacy and could direct physical therapy programs to correct these movements as initial conservative management could improve outcomes.

Movement differences between patients extend to other joints, with SD-LBP patients having significantly higher peak torque on the hip and knee compared to NS-LBP patients. Patients with spinal disorders often have hip dysfunction [19, 20], so the increased loads on the hip seen in SD-LBP patients may be related to a compensatory movement strategy to reduce loading on the lower back. Similarly, limitations in knee extension and emphasis on knee flexion have been proposed to be a compensatory mechanism for sagittal imbalance associated with LBP [21, 22]. Knowledge of load distribution and transmission along the kinetic chain in LBP patients may help shape how physical therapy programs target not only core and lumbar musculature, but also lower extremity muscle groups to improve function and alleviate pain and disability.

Examining the relationship between PROs and biomechanical compensatory movement could be important to understanding patient-specific differences in pain and disability, but there has been no clear established relationship between PROs and biomechanical movement thus far. In the literature, trunk muscle mass and the duration of time taken to complete a five-repetition sit-to-stand test have both been found to correlate with patient-reported outcomes of ODI and VAS [5, 23]. In surgical patients, thoracolumbar corrective fusion to realign sagittal vertical axis has been found to correlate with ODI outcomes suggesting a correlative relationship between SVA and ODI [15]. However, these studies do not establish a clear relationship between PROs and function or distinguish between patient groups. In our study, we found that torso velocity in both the vertical and anterior directions was found to weakly correlate with VAS and ODI, but dSVA and spine torque did not correlate with PROs within patient groups. Based on the literature and our findings, rigorous longitudinal collection of PROs is necessary to determine more correlative relationships between biomechanics and pain and disability and to determine the effectiveness of therapy.

The primary limitation of this study is the lack of a control group as we were unable to obtain STS metrics from age-matched controls. Comparing to controls could provide further insight into the differences between LBP patient groups. In relation to data presented on controls within the literature, we would expect LBP patients to have lower torso velocity and greater loading on the lower back [7], and less lumbar lordosis with the spine being in a more flexed postural alignment [24]. We would also expect the kinematics in patients with less severe symptoms, such as in the NS-LBP group, to be an intermediate between controls and patients with more severe symptoms, such as in the SD-LBP group. Obtaining data from age-matched controls could provide further insight into understanding compensatory movements in LBP patients, which can be used for targeted rehabilitation.

In conclusion, we found that different spinal disorders associated with LBP symptoms may relate to different compensatory strategies resulting in more or less load on the lumbar spine. The relationship between biomechanical compensation metrics, such as loading on the spine during the STS, did not associate with severity of symptoms across LBP patients. These findings could inform targeted rehabilitation approaches for specific spinal disorders and patient populations.
